# Advances in analyzing and engineering plant metabolic diversity

**DOI:** 10.1002/aps3.70017

**Published:** 2025-07-11

**Authors:** Kira J. Tiedge, Federico Roda, Stacey D. Smith, Gaurav D. Moghe

**Affiliations:** ^1^ Groningen Institute for Evolutionary Life Sciences University of Groningen Groningen The Netherlands; ^2^ Max Planck Tandem Group, Facultad de Ciencias Universidad Nacional de Colombia Bogotá Colombia; ^3^ Department of Ecology and Evolutionary Biology University of Colorado Boulder USA; ^4^ Plant Biology Section, School of Integrative Plant Science Cornell University Ithaca New York USA

The immense diversity of the members of the kingdom Plantae, so far comprising more than 400,000 known species (Guiry, 2024), is reflected not only in their morphological and genetic variability but also in their metabolic complexity. Plant metabolic networks are dynamic and expanding systems that evolve in a lineage‐specific manner and produce hundreds of thousands of structurally diverse metabolites, which can be broadly categorized into general (or core/primary) and specialized (or secondary) metabolites. General metabolites, such as carbohydrates, amino acids, and lipids, are essential for fundamental physiological processes like growth, development, and reproduction. In contrast, specialized metabolites like alkaloids, flavonoids, terpenoids, and other phenolic compounds often have an impact at different levels beyond central carbon metabolism—from allosteric regulation of proteins, subcellular organization, and intercellular interactions to organismal phenotypes, phylogeographic/interspecies diversification, biotic/abiotic interactions, and ecosystem maintenance (Weng et al., 2021; Ono and Murata, 2023).

The chemical diversity of plant metabolites constitutes a vast and largely untapped phytochemical space with significant potential for applications across multiple fields. In medicine, plant‐derived compounds have been a cornerstone of drug discovery for centuries. For example, alkaloids, like morphine and quinine, and terpenoids, such as paclitaxel, have revolutionized the treatment of pain, malaria, and cancer, respectively (Newman and Cragg, 2020; Atanasov et al., 2021). In agriculture, phytochemicals are increasingly recognized for their contribution to plant defense against pests and diseases, reducing the need for synthetic pesticides while promoting sustainable farming practices and food security (Sousa et al., 2021). Beyond medicine and agriculture, plant metabolites hold promise for applications in biotechnology and industrial processes. For instance, terpenoids and phenolic compounds are being investigated for their potential as biofuels, bioplastics, and natural food preservatives (Mewalal et al., 2017). Even though less than 10% of plant species have been thoroughly investigated for their chemical composition (Li and Vederas, 2009), it is estimated that plants produce over a million compounds, although pinpointing a specific number is challenging because of the heterogeneity of metabolite databases available (Wang et al., 2016; Nguyen‐Vo et al., 2020; Hawkins et al., 2021). Recent estimates suggest that the total number of unique structures across the entire plant kingdom likely spans into the millions to tens of millions (Engler Hart et al., 2025), indicating that over 99% of the phytochemical space remains unexplored and highlighting its vast and largely untapped potential.

To capture this broad range of metabolite diversity and function, a variety of techniques are used, such as specialized protocols for metabolite extraction, mass spectrometry, computational metabolomics including compound annotation, cheminformatics, bioassays, chemotaxonomy, phylogenomics, ancestral state reconstruction, and chemical ecology. As the revolutions in genomics, big data, and artificial intelligence (AI) have taken hold, there is an increasing need to develop high‐throughput alternatives for the above techniques and to leverage AI to address outstanding roadblocks. Similarly, there is a greater impetus to combine different “parts” (e.g., enzymes, regulators, transporters) for metabolic engineering and reconstruction of complex metabolic pathways. Nevertheless, as just one example of persisting challenges, the comparison of metabolomic datasets and experiments remains difficult, caused by differences in extraction and analytic methods. Furthermore, standards for the collection, preservation, and sharing of metabolomic data are only slowly evolving (Alseekh et al., 2021; Genesiska et al., 2024), and the ecological roles of many metabolites are still poorly understood (Kessler and Kalske, 2018), limiting our ability to harness their full potential.

In this special issue, we highlight a sampling of these approaches, providing more insights into plant metabolic diversity. A summary of the articles in these issues is provided below, broadly divided into novel bench techniques, data applications, and AI techniques. We anticipate that the applications of these methods will boost our understanding of the arsenal of chemical responses plants deploy to respond to their natural environments and will help efforts to breed/engineer these chemical traits into crops.

## Bench techniques and data generation

Plants contain a large diversity of compounds, many of which have medicinal properties. However, when these compounds are purified for bioassays or commercial applications, the large amounts of chlorophyll that are simultaneously extracted can present significant challenges. Existing solid‐phase extraction methods for separating chlorophylls can be expensive, cumbersome, or non‐specific, the latter resulting in alteration of the overall composition of the plant extract. In this special issue, Cavalloro et al. (2025) present a low‐cost, scalable solid‐phase extraction protocol that was able to remove >85% of the chlorophyll content with minimal changes to other tested classes of compounds. The authors tested this approach with leaves of 20 different plant species with medicinal potential and demonstrated good performance. The authors suggest that, given the reduced processing time, reduced waste generation, and high sample integrity, this protocol should be considered as an environmentally friendly approach to chlorophyll removal for bioactive compound discovery campaigns.

The transfer of biosynthetic genes between plant species is a powerful tool for metabolite production and the dissection of metabolic pathways, but the permanent expression of transgenes can interfere with normal plant growth. These detrimental effects can be alleviated by controlling gene expression through time and space using promoters that respond to specific wavelengths of light, a method known as optogenetics. In this issue, Lindström Battle and Sweetlove (2025) explore the use of the plant‐usable light‐switch elements (PULSE) optogenetic system in the liverwort *Marchantia polymorpha* L., a species increasingly used for plant metabolic engineering due to its dominant gametophytic growth. The system was successfully applied to control the production of poly‐3‐hydroxybutyrate (PHB), a biodegradable bioplastic, achieving similar PHB accumulation levels as constitutive expression but providing effective regulation with minimal leakage and negative effects. The findings represent a significant advancement in the metabolic engineering toolkit and provide a model for using optogenetic control in plant systems. As the authors discuss, this approach presents broader applications in plant metabolic engineering but also faces important challenges.

## Applications and data deployment

The history of plant metabolic evolution is rich in cases of convergence, such as the independent origins of caffeine biosynthesis in cacao, citrus, coffee, and tea (Huang et al., 2016). Gibson et al. (2025) examine the distribution of non‐proteogenic amino acids (NPAAs) across angiosperms using a combination of literature searching and new screening. While some NPAAs can act as amino acid analogs that can disrupt protein synthesis, these compounds can also serve defensive or signaling functions (Jander et al., 2020). Nevertheless, their extreme rarity has limited a deeper understanding of their functional significance. Gibson et al. show that lineages with NPAAs are widely distributed across the angiosperm tree, implying convergent origins, although they are particularly prevalent in legumes. This clustering of NPAA production in legumes suggests that this clade could serve as the model for dissecting the underlying pathway of NPAA synthesis and possibly explain trade‐offs among different NPAAs across species. More broadly, this work highlights the key role of tracing the evolution of biochemical traits across the phylogeny (“phylochemical mapping”) for identifying taxa likely to produce particular compounds and for localizing important lineages for reconstructing novel biosynthetic pathways.

Over the past decade, the ease of sequencing has resulted in a proliferation of genomic and protein sequences. If a researcher discovers a new gene involved in a metabolic process, the process of finding related genes, retrieving annotations from global repositories such as UniProt (https://www.uniprot.org/), performing alignment to find conserved/diverged residues, and creating figures can take significant time. The Computer‐Assisted Sequence Annotation (CASA) workflow, as presented by Takahashi et al. (2025), automates this manual process. This set of interconnected Python scripts can be run using a single wrapper script, from which parameters for each of the steps can be specified. CASA's modular nature also allows further modification and/or extension of this workflow. Moreover, this automated workflow generates publication‐quality vector graphics of sequence alignment. The authors describe the application of the CASA workflow for studying a putative cysteine protease from *Drosera capensis* L. (Cape sundew) and for forming testable mechanistic hypotheses for downstream experimental validation. The application of CASA in pathway discovery workflows may lead to significant time savings for biologists.

## AI for collecting and leveraging data

Yuan et al. (2025) provide a timely review of approaches for capturing metabolic diversity beyond traditional methods based on compound separation and fragmentation. Among the thousands of unique metabolites typically found in any given plant, only a small fraction (typically less than 10%) can be identified by comparison to reference spectra in databases or in‐house libraries (Bittremieux et al., 2022), leaving a vast body of unidentified and thus unstudied chemical diversity. Yuan et al. provide an accessible introduction to machine learning approaches that can classify peaks (e.g., as terpenoids, flavonoids, etc.) based on the features of known metabolites and, in some cases, even annotate the individual compounds. Moreover, the authors highlight the key questions that can be addressed by classifying compounds without precise annotation. For example, automated pipelines can be used to measure overall metabolic diversity and extract metabolic features to both characterize natural variation and test specific functional hypotheses. They give special attention to multivariate methods, such as discriminant analysis, which can be used to identify compounds that distinguish samples from different conditions or tissues, for example. With compelling empirical examples and a wealth of proposed applications, Yuan et al. underscore the great potential for better understanding the ecology and evolution of chemical diversity through the expansion of metabolic exploration beyond traditional annotation.

Open‐access databases are an integral part of the advancement of plant metabolic research, and the use of large language models (LLMs) can help to both fill and extract from these repositories. Here, Knapp et al. (2025) present ways in which LLMs can be used not only to expand existing databases by mining scientific articles but also to subset or filter existing databases based on complex linguistic patterns. They tested different prompt engineering strategies to extract validated enzyme–product and compound–species pairs and also developed a pipeline for translating images of tables (as can often be found in PDF files of scientific articles) into a machine‐readable format. By using these common tasks as benchmarking examples, the study provides a practical guide for plant researchers, demonstrating possible ways to apply modern AI tools in their research.

Overall, the articles in this special issue highlight the breadth of cutting‐edge approaches used for unraveling the diversity, function, evolution, and engineering of plant metabolism. The idea for this special issue emerged within the Phytochemical Section of the Botanical Society of America, which organized a symposium, “Standing your ground: Understanding plant defense from molecules to morphology,” held at the Botany 2023 meeting to strengthen the links between physiology, development, genetics, and chemistry for utilizing and engineering plant defensive mechanisms. With this special issue, we hope to further enforce these bonds by providing readers with new ideas and tools to explore and engineer the plant chemical space.

## AUTHOR CONTRIBUTIONS

K.J.T. and G.D.M. initiated this special issue, and S.D.S. and F.R. contributed to its development. All authors contributed to editorial duties for the manuscripts included in this special issue. All authors contributed text for the manuscript, and K.J.T. combined those contributions and led the writing and editing. All authors approved the final version of the manuscript.

## References

[aps370017-bib-0001] Alseekh, S. , A. Aharoni , Y. Brotman , K. Contrepois , J. D'Auria , J. Ewald , J. C. Ewald , et al. 2021. Mass spectrometry‐based metabolomics: A guide for annotation, quantification and best reporting practices. Nature Methods 18: 747–756. 34239102 10.1038/s41592-021-01197-1 PMC8592384

[aps370017-bib-0002] Atanasov, A. G. , S. B. Zotchev , V. M. Dirsch , International Natural Product Sciences Taskforce , and C. T. Supuran . 2021. Natural products in drug discovery: Advances and opportunities. Nature Reviews Drug Discovery 20: 200–216. 33510482 10.1038/s41573-020-00114-z PMC7841765

[aps370017-bib-0003] Bittremieux, W. , M. Wang , and P. C. Dorrestein . 2022. The critical role that spectral libraries play in capturing the metabolomics community knowledge. Metabolomics 18: 94. 36409434 10.1007/s11306-022-01947-y PMC10284100

[aps370017-bib-0004] Cavalloro, V. , A. Fossati , D. Rossi , S. Collina , and E. Martino . 2025. A practical and easy‐to‐scale protocol for removing chlorophylls from leaf extracts. Applications in Plant Sciences 13(4): e70018. 10.1002/aps3.70018 PMC12319715 40766902

[aps370017-bib-0005] Engler Hart, C. , Y. Gadiya , T. Kind , C. A. Krettler , M. Gaetz , B. B. Misra , D. Healey , et al. 2025. Defining the limits of plant chemical space: Challenges and estimations. GigaScience 14: giaf033. 40184432 10.1093/gigascience/giaf033 PMC11970369

[aps370017-bib-0006] Genesiska , J. Falcao Salles , and K. J. Tiedge . 2024. Untangling the rhizosphere specialized metabolome. Phytochemistry Reviews 2024: 1–11.

[aps370017-bib-0007] Gibson, M. , W. T. Santos , A. R. Oyler , L. Busta , and C. A. Schenck . 2025. A new spin on chemotaxonomy: Using non‐proteogenic amino acids as a test case. Applications in Plant Sciences 13(4): e70006. 10.1002/aps3.70006 PMC12319704 40766898

[aps370017-bib-0008] Guiry, M. D. 2024. How many species of algae are there? A reprise. Four kingdoms, 14 phyla, 63 classes and still growing. Journal of Phycology 60: 214–228. 38245909 10.1111/jpy.13431

[aps370017-bib-0009] Hawkins, C. , D. Ginzburg , K. Zhao , W. Dwyer , B. Xue , A. Xu , S. Rice , et al. 2021. Plant Metabolic Network 15: A resource of genome‐wide metabolism databases for 126 plants and algae. Journal of Integrative Plant Biology 63: 1888–1905. 34403192 10.1111/jipb.13163

[aps370017-bib-0010] Huang, R. , A. J. O'Donnell , J. J. Barboline , and T. J. Barkman . 2016. Convergent evolution of caffeine in plants by co‐option of exapted ancestral enzymes. Proceedings of the National Academy of Sciences, USA 11: 10613–10618. 10.1073/pnas.1602575113 PMC5035902 27638206

[aps370017-bib-0011] Jander, G. , U. Kolukisaoglu , M. Stahl , and G. M. Yoon . 2020. Editorial: Physiological aspects of non‐proteinogenic amino acids in plants. Frontiers in Plant Science 11: 519464. 33391293 10.3389/fpls.2020.519464 PMC7773597

[aps370017-bib-0012] Kessler, A. , and A. Kalske . 2018. Plant secondary metabolite diversity and species interactions. Annual Review of Ecology, Evolution, and Systematics 49: 115–138.

[aps370017-bib-0013] Knapp, R. , B. Johnson , and L. Busta . 2025. Advancing plant metabolic research by using large language models to expand databases and extract labeled data. Applications in Plant Sciences 13(4): e70007. 10.1002/aps3.70007 PMC12319720 40766897

[aps370017-bib-0014] Li, J. W.‐H. , and J. C. Vederas . 2009. Drug discovery and natural products: End of an era or an endless frontier? Science 325: 161–165. 19589993 10.1126/science.1168243

[aps370017-bib-0015] Lindström Battle, A. L. , and L. J. Sweetlove . 2025. Optogenetic control of transgene expression in *Marchantia polymorpha* . Applications in Plant Sciences 13(4): e11632. 10.1002/aps3.11632 PMC12319710 40766900

[aps370017-bib-0016] Mewalal, R. , D. K. Rai , D. Kainer , F. Chen , C. Külheim , G. F. Peter , and G. A. Tuskan . 2017. Plant‐derived terpenes: A feedstock for specialty biofuels. Trends in Biotechnology 35: 227–240. 27622303 10.1016/j.tibtech.2016.08.003

[aps370017-bib-0017] Newman, D. J. , and G. M. Cragg . 2020. Natural products as sources of new drugs over the nearly four decades from 01/1981 to 09/2019. Journal of Natural Products 83: 770–803. 32162523 10.1021/acs.jnatprod.9b01285

[aps370017-bib-0018] Nguyen‐Vo, T.‐H. , L. Nguyen , N. Do , T.‐N. Nguyen , K. Trinh , H. Cao , and L. Le . 2020. Plant metabolite databases: From herbal medicines to modern drug discovery. Journal of Chemical Information and Modeling 60: 1101–1110. 31873010 10.1021/acs.jcim.9b00826

[aps370017-bib-0019] Ono, E. , and J. Murata . 2023. Exploring the evolvability of plant specialized metabolism: Uniqueness out of uniformity and uniqueness behind uniformity. Plant & Cell Physiology 64: 1449–1465. 37307423 10.1093/pcp/pcad057 PMC10734894

[aps370017-bib-0020] Sousa, R. M. O. F. , A. C. Cunha , and M. Fernandes‐Ferreira . 2021. The potential of Apiaceae species as sources of singular phytochemicals and plant‐based pesticides. Phytochemistry 187: 112714. 33845406 10.1016/j.phytochem.2021.112714

[aps370017-bib-0021] Takahashi, G. R. , F. M. Cumpio , C. T. Butts , and R. W. Martin . 2025. The Computer‐Assisted Sequence Annotation (CASA) workflow for enzyme discovery. Applications in Plant Sciences 13(4): e70009. 10.1002/aps3.70009 PMC12319702 40766899

[aps370017-bib-0022] Wang, M. , J. J. Carver , V. V. Phelan , L. M. Sanchez , N. Garg , Y. Peng , D. D. Nguyen , et al. 2016. Sharing and community curation of mass spectrometry data with Global Natural Products Social Molecular Networking. Nature Biotechnology 34: 828–837. 10.1038/nbt.3597 PMC5321674 27504778

[aps370017-bib-0023] Weng, J.‐K. , J. H. Lynch , J. O. Matos , and N. Dudareva . 2021. Adaptive mechanisms of plant specialized metabolism connecting chemistry to function. Nature Chemical Biology 17: 1037–1045. 34552220 10.1038/s41589-021-00822-6

[aps370017-bib-0024] Yuan, X. , N. S. S. Smith , and G. D. Moghe . 2025. Analysis of plant metabolomics data using identification‐free approaches. Applications in Plant Sciences 13(4): e70001. 10.1002/aps3.70001 PMC12319716 40766901

